# Regional Anaesthesia in the Post-COVID Era

**DOI:** 10.5152/TJAR.2022.21433

**Published:** 2022-12-01

**Authors:** Yavuz Gürkan, Eren Yavuz Açıkalın, Umut Deniz Adanur, Mete Manici

**Affiliations:** 1 Department of Anaesthesiology and Reanimation, Koç University Faculty of Medicine, İstanbul, Turkey

**Keywords:** Brachial plexus blocks, COVID-19 complications, infraclavicular block, postoperative analgesia, regional anaesthesia

## Abstract

COVID-19 pandemic has changed clinical practice significantly. Trying to avoid airway manipulation to reduce viral transmission favoured regional anaesthesia techniques over general anaesthesia when feasible. In this case report, we share the anaesthetic care of a 45-year-old male with a history of intensive care unit admission due to COVID, positive pressure ventilation, and ECMO support. The patient was diagnosed with cubital tunnel syndrome, and his treatment was successfully applied as surgical release under infraclavicular block. This case is discussed in relation to recent trends in anaesthesiology, regarding patients with COVID-19 history.

**Main Points**

- COVID-19 altered anaesthesia preferences.
- Regional anaesthesia can provide surgical anaesthesia and/or analgesia for a great variety of patients undergoing surgery.
- We recommend avoiding general anaesthesia in patients with COVID-19 history.
- Interfascial plane blocks could be an alternative to neuraxial anaesthesia to provide analgesia.

## Introduction

COVID-19 has been a part of our lives since the start of the pandemic in early 2020.^1^ During this time, the disease itself and regulations to decrease transmission changed medical practice. These changes were mostly to decrease virus transmission during the medical care of patients. One of these changes was increased usage of regional anaesthesia procedures where it’s appropriate, as advised by both European and American authorities.^2^ One of the main goals was avoiding airway manipulations which was the main route of viral transmission, but it has other benefits as well. In patients with COVID-19 history, disease-related complications can affect the perioperative management of the patient.^3-6^ Using regional anaesthesia can be a solution to some of these problems. Peripheral,^7,8^ central nerve blocks,^8,9^ and interfascial plane blocks^10,11^ with their benefits in patients with COVID-19 history are discussed briefly. A sample case is used to better understand the situation. The case is an elective elbow surgery in a patient with a severe COVID-19 infection history requiring intensive care unit (ICU) admission and ECMO support before the operation.

## Case Presentation

This patient, a 45-year-old male without any known disease prior, was internalised because of dyspnoea due to COVID-19. During his stay, the patient required ICU admission and intubation following respiratory failure. His condition required continuous extracorporeal membrane oxygenation (ECMO) support for 14 days. He was discharged from ICU and then hospitalised after resolution of pulmonary inflammation. During his initial hospitalisation, sclerosing cholangitis and mediastinal fibrosis with superior vena cava syndrome were diagnosed, both thought to be secondary to COVID history. Patient was discharged with 0.6 mL subcutaneous enoxaparin twice daily.

During follow-up, the patient required intermittent hospitalisation due to pericholedoctal abscesses formed by sclerosing cholangitis. During his hospitalisation, the anticoagulant regime continued. Patient complained of pain in the left hand and was diagnosed with cubital tunnel syndrome by a hand surgery specialist. Routine painkillers and physical therapy were added to the patient’s treatment regime. Ulnar nerve release surgery in elective conditions was offered to the patient. Upon antibiotic treatment, patient’s complaints diminished, and laboratory tests and images confirmed that periductal abscess was resolved.

Following the resolution of abscess, surgery was planned with regional anaesthesia, following the informed consent of the patient. Surgery was planned with regional anaesthesia considering the patient’s COVID-19 history and comorbidities. Surgery was completed successfully with the left-sided infraclavicular block using ultrasound guidance. Following 2 mg of intravenous midazolam premedication, and sedation using propofol infusion 3 mg kg^−1^ per hour was used because of patient’s restlessness to increase patient comfort. Patient did not describe pain sensation after surgery, and the patient was discharged with the cure of his orthopaedic complaint.

## Discussion

Patients with a COVID-19 history present a challenge for anaesthesiologists because of disease complications. These complications are important for the choice of anaesthesia modality. Palpitations, chest pain, dysrhythmia, and decreased functional capacity can be seen following acute COVID-19 infection.^3^ General anaesthetic agents could increase the risk of cardiac dysfunction in patients with COVID-19 history.^12^ In pulmonary effects of disease, small airway disease and restrictive pattern functional limitation are found in some patients, 6 months following acute infection, even if the patient did not require oxygen supplementation.^4^ Hematologic complications include the risk of thromboembolic events, and risk remains increased, and median time of event is 78 days after acute infection.^5^ Thromboembolic events can occur as a complication of general anaesthesia as well.^12^ COVID-19-related thromboembolic event risk is higher in these patients, and some of them use anticoagulants.^13^ In such cases, both coagulation and bleeding-related complications can occur, and anaesthesiologists should be beware of them.

Choice of anaesthesia should involve all these considerations regarding COVID-19 history, as most of these complications can flare following general anaesthesia administration. Using regional anaesthesia avoids potential complications of general anaesthesia. Regional anaesthesia allows the patient to continue spontaneous respiration and diminishes the necessity for mechanical ventilation, preventing further barotrauma to the patient with a history of prolonged intubation, such as this patient.^6^ Other complications of general anaesthesia regarding cardiac function and thromboembolic events should be considered. Overall, avoiding general anaesthesia can prevent a variety of complications in patients with COVID-19 history. In postoperative settings, regional anaesthesia decreases analgesic requirement in various cases,^7,8^ avoiding side effects such as respiratory depression, dizziness, and hypotension related to postoperative opioids.

The type of regional anaesthesia should be selected case-wise. Peripheral nerve blockage is a popular choice of anaesthesia for surgeries regarding extremities.^7,8^ Variability of nerve block choices enables anaesthesiologists to personalise the regional anaesthesia technique according to patient needs and type of surgery. In this case, the surgical area was defined as the elbow joint; therefore, an infraclavicular block was used to achieve surgical anaesthesia.

Central neuraxial blocks can be used as the sole anaesthetic technique for caesarean sections, lower extremity surgeries, and abdominal surgeries.^8^ In limited cases, visceral surgeries can be completed with neuraxial blockade only,^9^ decreasing the necessity of general anaesthesia and avoiding potential complications arising from general anaesthesia. Anticoagulant usage is especially important for neuraxial procedures, as it is known to increase potential neurological complications. Spinal hematoma risk persists in patients with COVID-19 history because anticoagulants are a part of suggested treatment in patient with severe COVID-19 history who require hospital admission.

Interfascial plane blocks offer an alternative to epidural analgesia mainly for abdominal and thoracic surgeries,^10,11^ and their usage is increasing daily.^14,15^ Yet, these interfascial plane blocks provide mainly analgesia and not anaesthesia.^10^ These blocks are considered as part of multimodal analgesia and are not always enough to provide total pain-free period on their own.^7,10^ However, they reduce postoperative opioid requirement.^7,10,14^

## Conclusion

In accordance with The European Society of Regional Anaesthesia and Pain Therapy (ESRA)/The American Society of Regional Anesthesia and Pain Medicine (ASRA) societies recommendations, regarding post-COVID era, regional anaesthesia techniques present a great advantage for the safety of patients and help avoid general anaesthesia.^3^ For abdominal and thoracic surgeries, especially, interfascial plane blocks present an alternative to central neuraxial blocks and should be included in the armamentarium of anaesthesiologists to reduce postoperative opioid requirements.
